# Supplementary material for: Longitudinal and Cross-Sectional Relations Between Early Rise Time Discrimination Abilities and Pre-School Pre-Reading Assessments: The Seeds of Literacy Are Sown in Infancy
Source: Brain Sci. 2025 Sep 19;15(9):1012. doi: 10.3390/brainsci15091012 (PMC12468059; doi:10.3390/brainsci15091012)

Longitudinal and cross-sectional relations between early rise time discrimination abilities and pre-school pre-reading assessments: The Seeds of Literacy are sown in infancy

Supplementary Materials

Table S1. Parental performance on the screening battery.

Parental performance on the screening battery administered to determine infants' allocation to the family risk (FR) and family no risk (NFR) groups at the start of the SEEDS OF LITERACY project when infants were 5 months of age (scores for 88 parents of 44 children)

Screening Test	AR parents with dyslexia	AR parents without dyslexia	NAR parents
TOWRE[1]- Word reading subtest	88.1 (12.208)[ab]	109 (11.707)	109.348 (12.12)
TOWRE[1]- Non-word reading subtest	76.15 (28.856)[ab]	104.8 (12.722)	113.000 (8.613)
WIAT III[2] - Spelling subtest	85.575 (17.054) [ab]	106 (10.578)[c]	113.957 (8.952)
WIAT III[2] - Oral reading fluency	92.3 (12.868)[ab]	106.05 (7.423)	107.717 (7.833)
WIAT III[2] - Oral reading accuracy	86.65 (19.516)[ab]	105 (12.961)	109.438 (10.125)
WIAT III[2] - Oral reading rate	88.55 (14.013)[ab]	105.35 (7.707)	106.478 (6.659)
Woodcock-Johnson[3] - RAN	95.8 (11.496)	104.05 (11.455)	102.739 (14.543)
WAIS[4] - Digit span	12.4 (21.261)	14.65 (21.881)	11.783 (2.843)
WAIS[4] - Non-verbal IQ subtests	11.075 (2.267)[a]	12.1 (2.485)	13.000 (2.082)

[1]Test of Word Reading Efficiency (standardised score $M = 100$, $SD = 15$); [2]Wechsler Individual Achievement Test (standardised score $M = 100$, $SD = 15$); [3]Woodcock-Johnson test (standardised score $M = 100$, $SD = 15$); [4]Wechsler Adult Intelligence Scale (standardised score $M = 10$, $SD = 3$)
One-way ANOVA with post-hoc Tukey tests: [a]FR with dyslexia < NFR; [b]FR with dyslexia < FR without dyslexia, [c]FR without dyslexia < NFR.

Figure S1. Detailed breakdown of the sample sizes included in the analyses.

Detailed breakdown of the sample sizes included in the analyses for the 10-month and 60-month Rise Time (RT) discrimination tasks, and their composition based on children's original family risk status (determined based on parental screening when the children were 5 months) and children's performance in the 60-month screening battery.

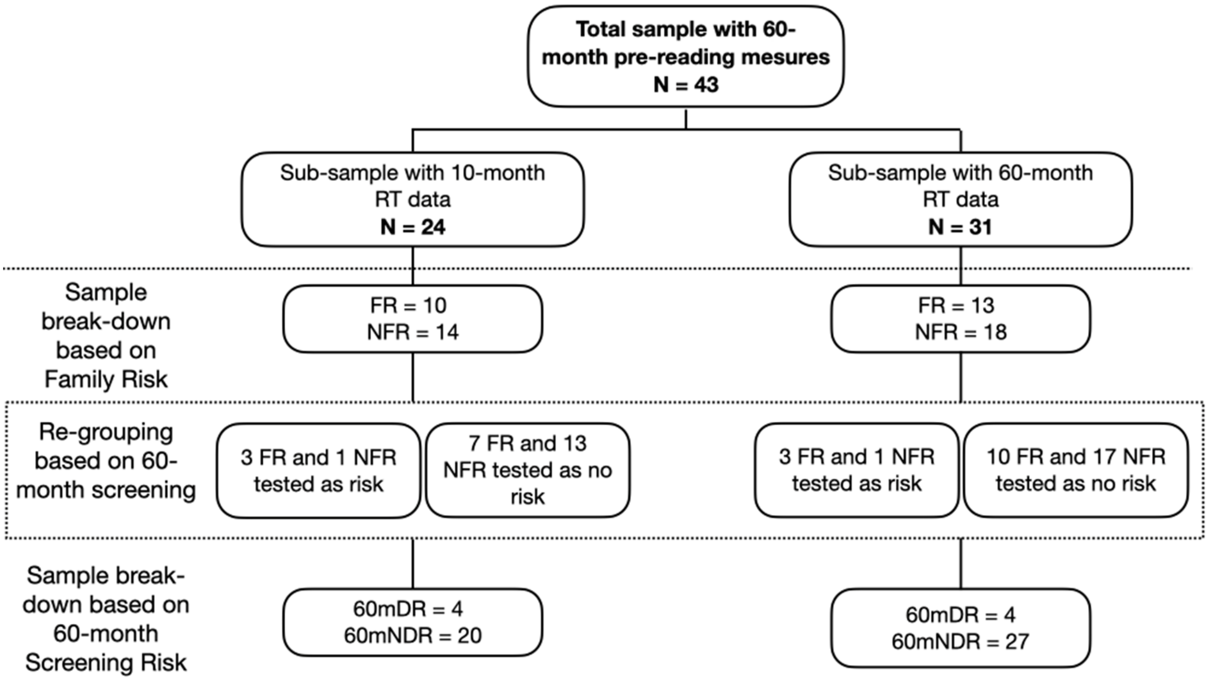

Figure S2. Distribution of 60-month Rise Time discrimination data.

Preliminary inspection of the 60-month Rise Time discrimination data indicated that the discrimination thresholds (msec) were severely skewed and were unsuitable for parametric analyses. The thresholds were rank normalised using the rank.normalize function in R. The figure below presents the Rise Time threshold distributions at 60 months before (A) and after rank normalisation (B).

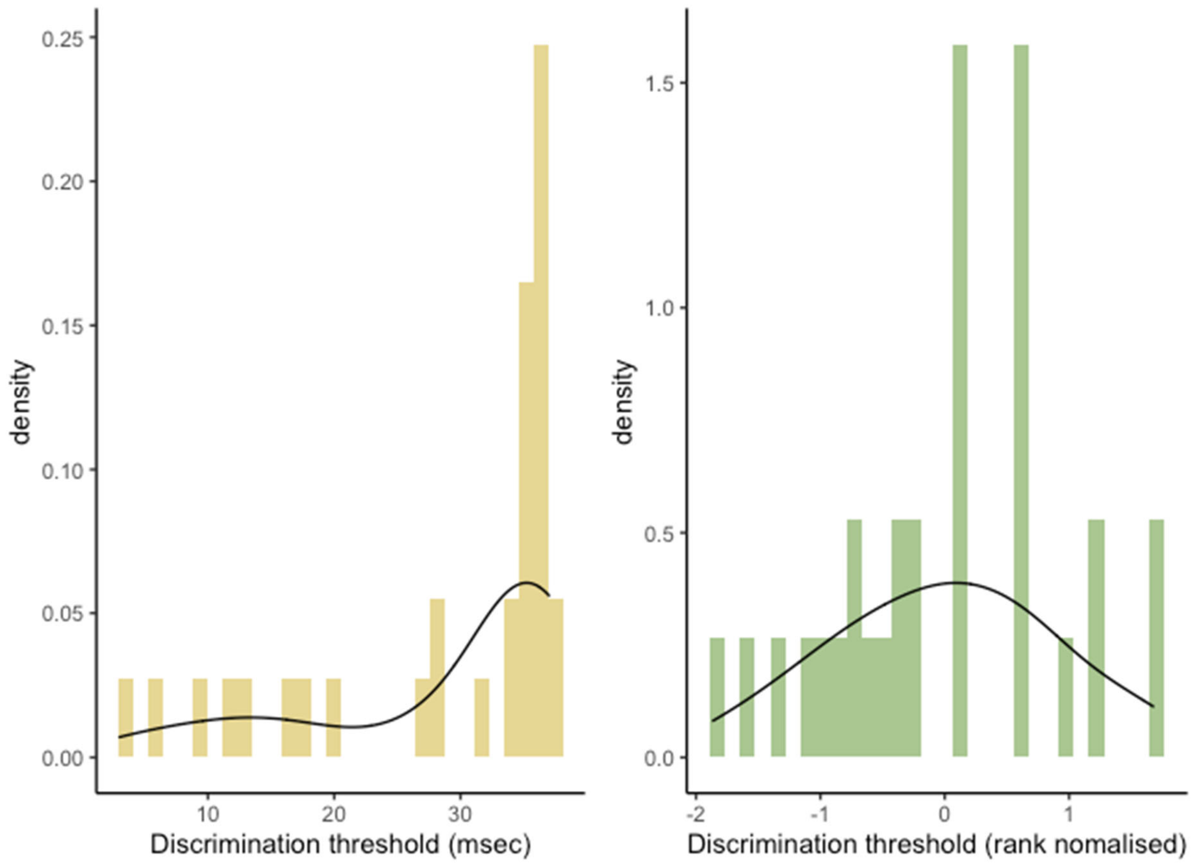

Supplement: Supplementary file 1 [file brainsci-15-01012-s001.zip › brainsci-3816693-supplementary.pdf]
